# Impact of the body mass index on perioperative immunological disturbances in patients with hip and knee arthroplasty

**DOI:** 10.1186/s13018-017-0557-4

**Published:** 2017-04-08

**Authors:** Simon Jasinski-Bergner, Anna-Luise Radetzki, Janine Jahn, David Wohlrab, Heike Kielstein

**Affiliations:** 1 Department of Anatomy and Cell Biology, Faculty of Medicine, Martin Luther University Halle-Wittenberg, Grosse Steinstrasse 52, 06108 Halle (Saale), Germany; 2 Department for Orthopedics, Trauma and Reconstructive Surgery, University Hospital of Halle (Saale), Halle (Saale), Germany

**Keywords:** Obesity, NK cells, Knee joint, Hip joint, Artificial joint implantation

## Abstract

**Background:**

Obesity increases the risk for knee and hip joint implantation and negatively contributes to wound healing. In this study, in 52 patients undergoing hip and knee arthroplasty the amount of peripheral immune effector cells pre- and post-operative, as well as the expression of certain soluble factors affecting the functions of immune effector cells were investigated.

**Methods:**

The peripheral immune cells and the expression of the soluble factors were determined by flow cytometry and correlated to each other in dependency of the BMI, the sex, and the kind of arthroplasty.

**Results:**

The pre-operative amounts of peripheral NK cells and cytotoxic T cells significantly decreased with increasing BMI. Furthermore, the expression of the immunomodulatory adipokine leptin nicely correlated to the BMI. These effects were stronger in males than in females. Furthermore, the correlation of the activation marker sTNF-R and peripheral T cells strongly decreased with increasing BMI. While IL-6, CD40L, and MPO were significantly induced after surgery, there were no correlations to the BMI.

**Conclusions:**

The known wound-healing problems in obese patients and the osteoarthritis per se can be linked to the BMI. While obese patients exerted reduced peripheral NK cells and cytotoxic T cells (CTLs), IL-6 showed no involvement. However, the adipokine leptin strongly increased with the BMI strengthening its role as immunomodulatory molecule negatively interfering the functions of immune effector cells.

## Background

Overweight (body mass index (BMI) ≥25 kg/m^2^) and obesity (BMI ≥ 30 kg/m^2^) strongly increase among the western world population [1]. Indeed, recent studies demonstrated the correlation between obesity and the prevalence of the arthrosis mediated need for an artificial hip and knee joint implantation [2]. Furthermore, it could be demonstrated that obesity is linked with postoperative wound healing complications of hip and knee arthroplasty [3]. Also, a significant correlation of obesity and the expression of TNF-α, CRP, and IL-6 has been described in literature [4]. In the last decades, the number of total hip and knee replacements increased worldwide. Obesity is one of the leading key factors for development of osteoarthritis as well as for survivorship of hip and knee arthroplasties.

White adipose tissue, mainly composed of adipocytes, is not only an energy reservoir or a protective envelope located subcutaneously and around the internal organs (visceral adipose tissue), it further secretes certain cytokines, so-called adipokines, involved in important biological processes including immunology and metabolism [5].

Adipokines can exert anti- and pro-inflammatory effects. Leptin is the most important immunoregulatory adipokine. Apart from its T-lymphocyte modulating role [6], it also inhibits the proliferation of monocytes and macrophages and enhances their cytokine secretion [7, 8]. Our group could demonstrate that natural killer (NK) cells express the functional isoform of the leptin receptor (Ob-Rb) [9] and a short-term stimulation of NK cells with leptin resulted in an increased interferon (IFN)-γ secretion and tumor cell lysis [10]. Obese individuals have highly elevated serum adipokine and cytokine levels—a so-called chronic low-grade inflammation. Thus, a long-term incubation of NK cells with leptin (as seen in obese individuals) impairs the anti-tumoral NK cell functions [10], an interesting point, because obesity increases the risk for certain tumor diseases, like colorectal cancer, renal cancer, post-menopausal breast cancer, and prostate cancer [1]. The most highly expressed mRNA in adipocytes of normal weight individuals is the one of the anti-inflammatory adipokines, the adiponectin [11]. Adiponectin is inversely correlated to plasma CRP levels [12]. Furthermore, adiponectin production in adipocytes is inhibited by the pro-inflammatory cytokines tumor necrosis factor (TNF)-α and interleukin (IL)-6 [13], which are also produced in fat tissue next to resistin, visfatin and many others.

To investigate the link between obesity and the immune status in regard of known wound-healing problems in patients undergoing primary hip and knee arthroplasty pre- and post-operatively, acquired blood samples were investigated and analyzed for immune effector cells, selected adipokines, and certain clinical markers. The results were compared between pre- vs. post-operative status, hip vs. knee joint implantation, male vs. female, and normal weight vs. overweight vs. obesity.

## Methods

### Study population

The study population includes 52 volunteers (25 males and 27 females) aged between 40 and 80 years. These volunteers were patients in the Department for Orthopedics, Trauma and Reconstructive Surgery, University Hospital, Halle (Saale), Germany receiving a primary hip or knee joint implantation. From the males, 10 patients received a total knee arthroplasty and 15 patients received a total hip replacement, while from the female group, 16 patients received a knee and 11 patients received a hip implantation.

The study population was further subdivided according to the body mass index (BMI) into the following groups: normal weight (BMI <25 kg/m^2^), overweight (25 kg/m^2^ ≤BMI< 30 kg/m^2^), and obese (BMI ≥30 kg/m^2^). For an exact summary of the subdivisions of the study population and the respective age, please see Table 1.

**Table 1:** Overview of the patients who participated in this study

	Male			Female		
Subdivision by weight	Normal weight	Overweight	Obese	Normal weight	Overweight	Obese
	*n* = 10 (63 years)	*n* = 9 (70 years)	*n* = 6 (69 years)	*n* = 7 (71 years)	*n* = 9 (67 years)	*n* = 11 (67 years)
Subdivision by joint implantation	Knee	Hip		Knee	Hip	
	*n* = 10 (63 years)	*n* = 15 (69 years)		*n* = 16 (69 years)	*n* = 11 (68 years)	
Total	*n* = 52 (67 years)					

Mean age in brackets

Exclusion criteria were denial of the declaration of consent, non-joint-related proven centers of infection (chronic or acute), known metabolic or endocrine diseases (e.g., diabetes mellitus type I, hypo- or hyper-thyreoses), and immune suppression (congenital or acquired).

This study followed the Declaration of Helsinki and was approved by the local ethics committee of the Faculty of Medicine, Martin Luther University Halle-Wittenberg, Halle (Saale), Germany.

### Sample collection and preparation

Fasting blood samples were collected routinely pre- (3 ± 2 days) and post-operatively (5 ± 2 days). From each donor, 2.7 ml of heparinized blood was used for further analyses. Quantification of CRP and leukocytes was also performed routinely.

### Flow cytometry

To determine and quantify various immune effector cell populations (CD3^−^ CD20^+^ B cells; T cells including CD3^+^ CD4^+^ T helper cells and CD3^+^ CD8^+^ cytotoxic T cells; CD3^+^ CD56^+^ NKT cells and CD3^−^ CD56^+^ NK cells) in the blood samples, flow cytometry was performed at a LSRFortessa unit (Becton Dickinson, BD, Franklin Lakes, NJ, USA). The following murine monoclonal antibodies were applied: PE-Cy7 anti-human CD3 [UCHT1] (BD), APC anti-human CD4 [L200] (BD), PE anti-human CD8 [HIT8a] (BD), APC-H7 anti-human CD20 [2H7] (BD), and APC anti-human CD56 [NCAM16.2] (BD). Prior to this, the erythrocytes were removed by hypotonic lysis.

### Cytokine analysis

Cytokines and soluble factors were determined by usage of the FlowCytomix human obesity 9plex kit (eBioscience, San Diego, CA, USA) according to the manufacturers’ protocol. This kit allows an antibody-based detection and quantification of soluble CD40L, soluble ICAM1, IL-6, Leptin, MCP-1, MPO, OPG, resistin, and soluble TNF-R. After incubating the serum with the respective capture antibodies linked to beads, a biotinylated second antibody was added. In the last step, PE labeled streptavidin was added for emitting the fluorescent signal. The respective concentrations were determined by usage of included standards for each target.

### Statistical analyses

Microsoft Excel 2010 (Microsoft Corporation) and SPSS 16.0 (IBM) were used to calculate mean and standard derivation or to perform the *t* test. For the two-sided *t* test, unequal variances have been selected and Levene’s test was performed. Significance was assigned when *p* < 0.05 and marked with a star (or if lower than 0.005 with two stars).

## Results

### Comparison of the pre- and post-operative immune status of patients undergoing hip and knee arthroplasty

**Fig. 1:**
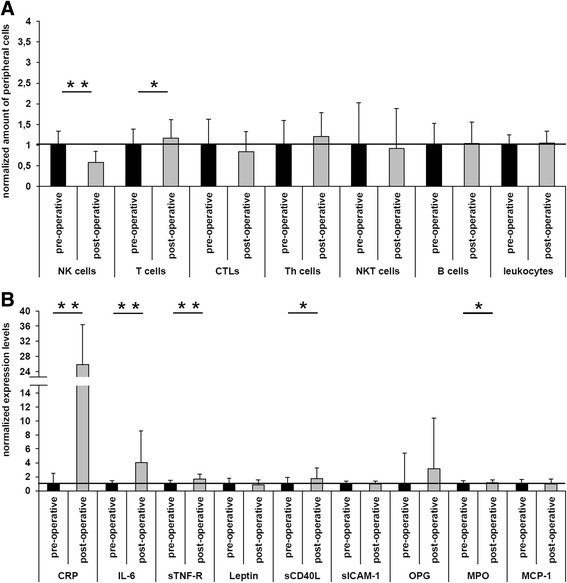
Pre- and post-operative comparative analysis of immunological parameters in patients undergoing hip and knee arthroplasty. **a** The amount of peripheral immune effector cells in the blood samples of 52 patients receiving hip and knee arthroplasty were determined pre- and post-operatively by flow cytometry. While NK cells were statistically significantly reduced after surgery, the T cells were significantly increased. From further analyses of Th cells, CTLs and NKT cells can be concluded that this induction is mainly caused by an increase of Th cells. **b** The differential expression of several soluble factors was pre- and post-operatively determined in the blood samples of 52 patients by flow cytometry and the usage of the FlowCytomix human obesity 9plex kit (eBioscience) revealing a statistically significant induction in the serum level of CRP, IL-6, sTNF-R, sCD40L, and MPO after surgery

A statistical significant decrease of NK cells (*p* < 0.005) after the surgical intervention (Fig. 1a) could be detected. Furthermore, a significant increase of peripheral T cells (*p* < 0.05) was observed post-operatively, most likely caused by an increase of T helper (Th) cells, since a decreased amount of cytotoxic T cell (CTL) and not affected NKT cells were measured (Fig. 1a). Additionally, no differences in the presence of B cells and leukocytes were detected.

In addition to the analysis of the lymphocyte subsets, also the expression of certain immunological relevant molecules including adipokines was determined. The CRP levels increased statistically significant (more than 26 times; *p* < 0.005; Fig. 1b) after the surgical procedure. Also, the pro-inflammatory cytokine IL-6 was significantly increased (about four times; *p* < 0.005; Fig. 1b). As part of the post-operative status of immune activation, sTNF-R was also significantly increased (*p* < 0.005). Finally, also the levels of sCD40L (*p* < 0.05) and myeloperoxidase (MPO; *p* < 0.05) were significantly enhanced after the surgical intervention. However, this increase was very modest (sCD40L: 1.74 times and MPO: 1.17 times). Other measured parameters showed no significant differences in their expression pattern when comparing the pre- and post-operative status (Fig. 1b).

### Differential amounts of peripheral immune effector cells in dependency of patient parameters

**Fig. 2:**
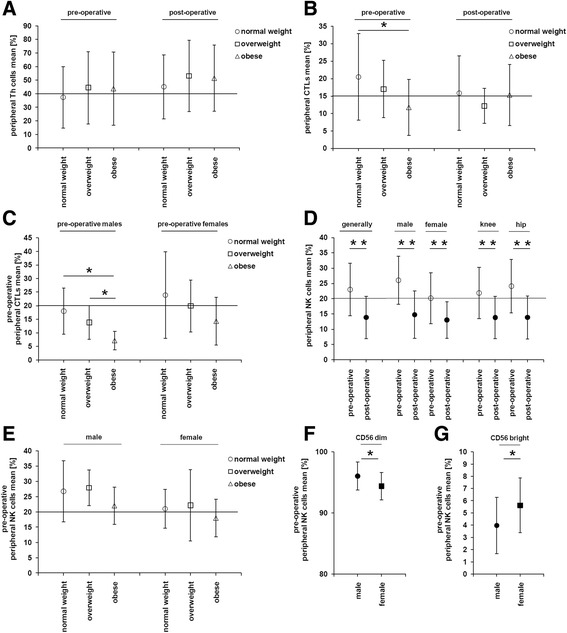
Pre- and post-operative immunoscoring of selected peripheral immune effector cells in relation to the body weight. The results of the determined immune effector cells in the blood samples of the 52 patients by flow cytometry were correlated with certain parameters including body weight, sex, and kind of arthroplasty. The values are expressed in % of the PBMCs, and in the case of CD56bright and CD56dim NK cells, the values are expressed in % of the NK cells. **a** The amount of peripheral Th cells increased with the BMI in males and females, but not statistically significant. **b** In the pre-operative condition, the amount of CTLs was statistically significantly reduced in obese patients, when compared to normal weight patients. This effect could not be observed directly after surgery. **c** In the pre-operative phase, the number of peripheral CTLs negatively correlated to the BMI in males and females. These effects were only statistically significant in males. **d** The by far strongest effects were determined for NK cells. In the constellations male vs. females and knee vs. hip joint implants, the NK cells were significantly reduced after surgery. **e** The number of peripheral NK cells decreased with the BMI in males and females. However, these effects were weak and not statistically significant. Interestingly, in the pre-operative phase, males had significant higher levels of CD56^dim^ NK cells than females (**f**), but for CD56^bright^ NK cells, it was the other way around (**g**)

No differences could be observed when comparing B and NKT cells pre- vs. post-operatively, male vs. female, knee vs. hip joint implantation, or normal weight vs. overweight vs. obese. Contrary to that, Th cells were determined at a higher percentage in the blood samples of overweight and obese patients when compared to the normal weight patients. This effect could be observed pre- and post-operatively. However, the differences were not statistically significant (Fig. 2a).

On the other hand, the pre-operative CTLs were statistically significantly decreased in obese patients as compared to normal weight patients (Fig. 2b). Interestingly, this effect was more pronounced in males than in females (Fig. 2c). No noteworthy differences of the peripheral CTLs could be observed in the other comparisons.

The by far strongest differences could be detected in the quantities of peripheral NK cells. While male patients showed the highest presence of peripheral NK cells pre- and post-operatively, no differences were observed when comparing knee vs. hip joint implantations. All differences concerning the number of peripheral NK cells in pre- and post-operative groups were statistically significant, demonstrating the general effect of the post-operative decline of peripheral NK cells and herewith an impairment of one part of the innate immune system (Fig. 2d).

In analogy to the pre-operative status of CTLs, also the pre-operative NK cells were decreased in obese patients when compared to normal weight patients both in males and females. However, the difference failed to reach statistical significance (Fig. 2e).

As already mentioned, numbers of peripheral NK cells were higher in males as compared to females. Further investigations demonstrated that these NK cells were NK cells with a low expression of CD56 (CD56^dim^). Interestingly, numbers of CD56^bright^ NK cells were significantly higher in females than in males (Fig. 2f, g). While CD56^dim^ NK cells represent 90% of the peripheral blood NK cells and reveal a higher cytotoxicity, the CD56^bright^ NK cells are more efficient cytokine producers like IFN-γ.

### Correlation between CRP level and BMI in females

**Fig. 3:**
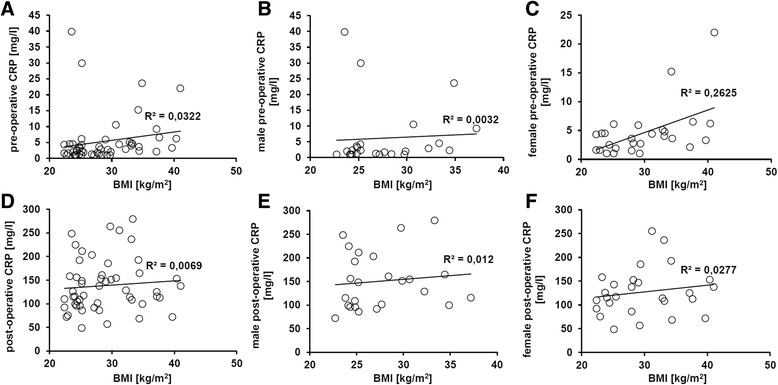
Correlation between CRP levels and BMI in females and males. The CRP levels were determined in the blood samples of 52 patients of hip and knee arthroplasty routinely pre- and post-operative. **a**–**c** In the pre-operative phase, there was a correlation between CRP levels and the BMI. However, this effect was just observed in females, but not for males. This effect was lost directly after surgery due to the massive increase of CRP level per se (**d**–**f**)

The acute-phase reactant CRP is a marker for inflammation and is induced by IL-6. CRP and IL-6 are pro-inflammatory mediators secreted among others by adipose tissue. Therefore, the pre- and post-operative plasma level of CRP was correlated with the BMI of the patients. In females, the pre-operative plasma level of CRP tends to correlate with the BMI, while in males, no correlation could be detected (Fig. 3a–c).

The post-operative CRP plasma levels dramatically increased in all patients due to surgery when compared to the pre-operative status (*p* < 0.05). No further correlation to the BMI could be observed after surgery (Fig. 3d–f).

### BMI-linked leptin expression correlates strongest in knee joint implantations and in males

**Fig. 4:**
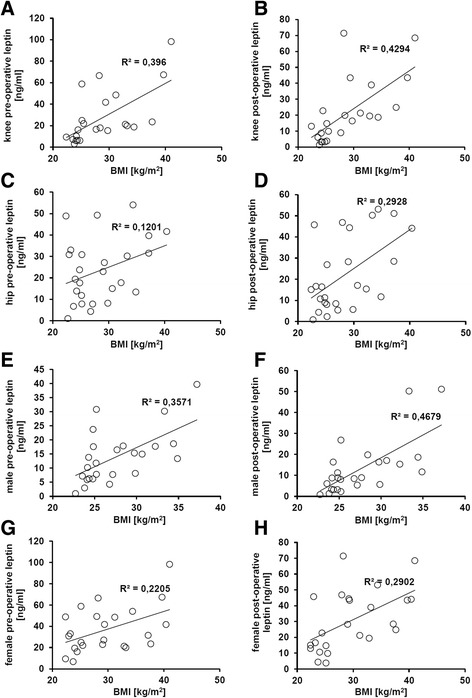
BMI-linked leptin expression. **a**–**h** The leptin serum levels of the 52 patients were correlated to the BMI in dependency of the kind of performed arthroplasty and in dependency of the sex. The leptin expression levels correlated nicely to the BMI pre- and post-operatively. However, the effect was stronger in knee joint implantation than in hip joint implantation and was stronger in males when compared to females

The expression of leptin nicely correlated to the BMI. This correlation was stronger in knee joint implantations than in hip joint implantations (pre- and post-operative; Fig. 4a–d).

Furthermore, the correlation between leptin expression and BMI was stronger in males than in females (pre- and post-operative; Fig. 4e–h). The other determined soluble factors were also correlated to the BMI, and the results are summarized in Table 2.

**Table 2:**
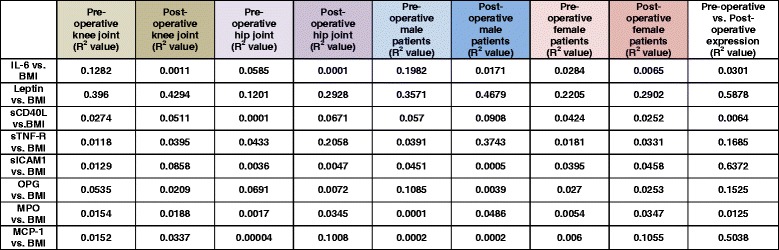
The expression of determined soluble factors in dependency of the BMI in the blood sera of the patients

### Correlation between peripheral T cells and sTNF-R expression in dependence of the BMI

**Fig. 5:**
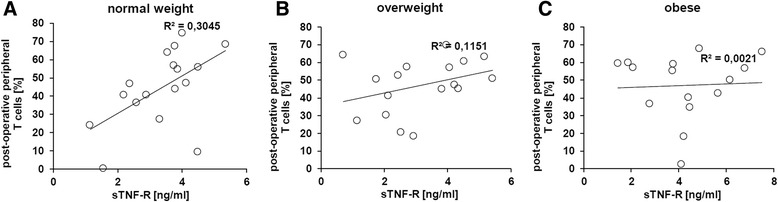
Correlation between post-operative peripheral T cells and sTNF-R expression in dependence of the body weight. **a**–**c** The correlation of serum levels of the activation marker sTNF-R with the amount of peripheral T cells decreased strongly with increasing BMI

Figure 2c demonstrates a decreasing amount of peripheral CTLs with increasing BMI of the patients. This effect was detectable in both sexes, with significant results in males. Furthermore, Fig. 1b shows a significant increase of the expression of the early inflammation marker sTNF-R after the surgical procedures. It was also the question addressed whether the sTNF-R expression shows any correlation with the BMI. Indeed, from normal weight to overweight to obese, the correlation between post-operative peripheral T cells and sTNF-R expression dramatically decreases (Fig. 5a–c), which can be explained by a declining T cell activity with an increasing BMI.

## Discussion

In this study, the pre- and post-operative immune status with a special focus on the BMI of patients receiving knee and hip arthroplasty was investigated. Indeed, the post-operative peripheral NK cells were statistically significantly (*p* < 0.005) reduced when compared to the pre-operative status. The cytokines IL-15 and IL-2 can induce proliferation in NK cells [14]. While IL-2 is mainly expressed in Th cells, IL-15 is constitutively expressed by certain immune cell populations including monocytes, macrophages, and dendritic cells [15]. In the blood, circulating monocytes are recruited to inflamed tissues and can derive to macrophages or dendritic cells [16]. The removal of the inflammatory site due to the arthroplasty could therefore explain the post-operative reduction of peripheral NK cells. Another in general explanation for that effect has been described as “suppression of cellular immunity after surgical stress” leading to a downregulation of Th cells, cytotoxic T cells (CTLs), and natural killer cells [17].

In contrast to that, a modest but statistically significant (*p* < 0.05) post-operative increase of peripheral T cells was detected; while CTLs and NKT cells were downregulated, the Th cells were induced. However, the reported effect of suppression of cellular immunity after surgical stress [17] has been reported for until at least 2 weeks post-operatively. In this study, the post-operative blood samples were taken 5 ± 2 days after surgery.

The post-operative increase of T cells would also explain the detected post-operative-enhanced secretion of IL-6, which is among others secreted by T cells [18], and the soluble form of CD40L, which is shed from the surface of activated T cells [19].

Analysis of peripheral blood lymphocyte subsets has become an essential tool in the evaluation of immunological and pathological disorders. By analyzing the composition of the immune effector cells, it turned out that the amount of pre-operative peripheral NK cells and CTLs is declining from normal weight to overweight and to obese patients. Indeed, that effect was stronger in males than in females. Males even exerted higher amounts of CTLs and NK cells per se. Such gender-related differences in the amount of CTLs and NK cells are in line with the reference values for lymphocyte subsets in Caucasians [20, 21].

Overweight and obesity increase the formation of osteoarthritis [22], negatively influence wound-healing processes [23], and even increase the prevalence of certain tumor diseases [1]. In prior studies, it was demonstrated that long-term effects of the adipokine leptin led to immunomodulatory functions on various immune cells including NK cells [10]. Due to the fact that leptin expression correlates with the amount of adipose tissue [24], the immunomodulatory effects of long-term leptin incubation on immune effector cells could be an explanation for the reduced wound healing in overweight and obese patients.

Indeed, in this study group, a correlation of leptin expression and the BMI could be observed. The strongest leptin correlation to the BMI occurred in the subgroup of males and in the subgroup receiving a knee joint implantation.

However, literature reports higher physiological plasma leptin level for women [25], a fact that not necessarily disagrees with the observation from this study, investigating a patient cohort undergoing hip and knee arthroplasty. Interestingly, a study with a large cohort demonstrated that plasma leptin levels are stronger linked to a cardiovascular death in males than in females [26]. Furthermore, it could be shown that high-elevated leptin levels in obese individuals result in the development of a leptin resistance [10].

The data from this study demonstrate for the first time that numbers of peripheral CTLs and NK cells are decreased in pre-operative obese patients when compared to normal weight patients suffering from osteoarthritis. NK cells usually form 5 to 15% of the peripheral blood mononuclear cells [27]. A decreased amount of peripheral NK cells has been reported for rheumatoid arthritis [28]. Interestingly, in osteoarthritis, NK cells highly infiltrate the synovial-tissue [29] offering an explanation for the determined decrease of peripheral NK cells and CTLs in obese patients with a more distinct inflammation and worsened wound-healing processes per se. It is furthermore noteworthy that in obese patients, the amount of circulating regulatory T cells is reduced, a circumstance that promotes inflammational processes.

Osteoarthritis is simply defined as non-inflammatory arthritis in comparison to rheumatoid arthritis. However, this old-fashioned classification has been described as very unfortunate in the actual literature [30]. Naturally, osteoarthritis occurs with inflammational processes due to cartilage and synovial destruction [30] in notable sized areas (for example hip and knee) when compared to the sizes of certain tumor diseases. Actual literature defines osteoarthritis not as one disease but rather a consequence of many predisposing factors like age, joint trauma, and obesity [30]. Interestingly, most frequently infiltrating immune effector cells are among other T cells. However, the absolute number of infiltrating immune effector cells is lower than in rheumatoid arthritis [31]. Therefore, certain parameters involved in immune status and wound healing were determined, including peripheral immune cells and selected soluble plasma factors.

The early inflammation marker sTNF-R is produced for example by activated T cells and is upregulated in synovial T cells of rheumatoid arthritis patients [32]. Indeed, in this study, it could be demonstrated that the expression of sTNF-R in normal weight patients is correlated to the amount of peripheral T cells. This correlation strongly decreases with increasing BMI, leading to the hypothesis of impaired functions of peripheral T cells in obese patients, next to the fact of the reduced amount of peripheral T cells in obese patients per se. However, further studies are necessary to determine such impaired T cell functions in obese humans when compared to normal weight persons.

## Conclusions

In dependency of the BMI, a reduced amount of peripheral NK cells and CTLs as well as a reduced expression of activation markers were observed, while the leptin levels increased with the BMI. This demonstrates some of the consequences of obesity on the immune system potentially negatively contributing to known wound-healing problems in obese patients.

## Abbreviations

BMI: Body mass index
CRP: C-reactive Protein
CTL: Cytotoxic T cells
e.g.: Exempli gratia
IL-6: Interleukin 6
MCP-1: C-C motif chemokine ligand 2
MPO: Myeloperoxidase
NK cell: Natural killer cells
OPG: Tumor necrosis factor receptor superfamily member 11b
Th cell: T helper cell
TNF-R: Tumor necrosis factor receptor superfamily member 1A
TNF-α: Tumor necrosis factor α
vs.: Versus
